# Telomerase in Brain: The New Kid on the Block and Its Role in Neurodegenerative Diseases

**DOI:** 10.3390/biomedicines9050490

**Published:** 2021-04-29

**Authors:** Gabriele Saretzki, Tengfei Wan

**Affiliations:** Biosciences Institute, Campus for Ageing and Vitality, Newcastle University, Newcastle upon Tyne NE4 5PL, UK; Tengfei.wan@ncl.ac.uk

**Keywords:** telomerase, TERT protein, brain, neuron, neurodegenerative disease, autophagy

## Abstract

Telomerase is an enzyme that in its canonical function extends and maintains telomeres, the ends of chromosomes. This reverse transcriptase function is mainly important for dividing cells that shorten their telomeres continuously. However, there are a number of telomere-independent functions known for the telomerase protein TERT (Telomerase Reverse Transcriptase). This includes the shuttling of the TERT protein from the nucleus to mitochondria where it decreases oxidative stress, apoptosis sensitivity and DNA damage. Recently, evidence has accumulated on a protective role of TERT in brain and postmitotic neurons. This function might be able to ameliorate the effects of toxic proteins such as amyloid-β, pathological tau and α-synuclein involved in neurodegenerative diseases such as Alzheimer’s disease (AD) and Parkinson’s disease (PD). However, the protective mechanisms of TERT are not clear yet. Recently, an activation of autophagy as an important protein degradation process for toxic neuronal proteins by TERT has been described. This review summarises the current knowledge about the non-canonical role of the telomerase protein TERT in brain and shows its potential benefit for the amelioration of brain ageing and neurodegenerative diseases such as AD and PD. This might form the basis for the development of novel strategies and therapies against those diseases.

## 1. Introduction

Telomerase is a very ancient and versatile enzyme in eukaryotes. Discovered originally in protozoans by Nobel Laureate Elizabeth Blackburn, its importance for human health during development, ageing and cancer soon became obvious. While the main function of telomerase is the maintenance of telomeres, additional functions of the protein component TERT were discovered in recent years.

A protective function of TERT has been established in human and mouse brain, but there are still many open questions. The review aims to summarise the current progress in this rapidly developing field. A particular emphasis is directed towards the potential benefit to alleviate neurodegeneration by increasing the physiological levels of TERT using either natural or synthetic telomerase activators as well as adenoviral overexpression.

## 2. Canonical and Non-Canonical Functions of Telomerase

Telomerase an enzyme well-known for elongating telomeres in dividing cells. It consists of two main components: the protein TERT (Telomerase Reverse Transcriptase) with reverse transcriptase function and an RNA component (TR: Telomerase RNA or TERC: Telomerase RNA Component) which contains the template for telomere synthesis de novo. This telomere-maintaining function is the canonical role of telomerase and is also called “telomerase activity” (TA). Although the two main components are sufficient for telomerase activity in vitro [1] in cells the enzyme is associated with a number of different proteins binding either the RNA component (for example dyskerin, NOP10, La, TCAB1 and others) or the TERT protein (Hsp90, pontin, reptin). These are important for the regulation of telomerase assembly and activity [2].

Telomerase is present in almost all eukaryotic organisms, including such primitive species as sponges [3], yeast (*S. cerevisiae, Schizosacharomyces pombe*) [4] nematodes (*C. elegans*) [5] and lobsters (where it might be responsible for lifelong growth and remarkable longevity) to name just a few [6]. In higher eukaryotes including plants [7] it is active at least at certain developmental stages or in selected adult cells and tissues. However, there are also exceptions within eukaryotes, for example in insects like diptera (for example *Drosophila*) where telomerase is absent, and telomeres are maintained via retrotransposons [8].

Even among mammals, there are significant differences in telomere and telomerase biology. While telomerase in rodents is active during adult life in most somatic cells, in humans it is downregulated early during development and is only active in germline cells, stem cells, endothelial cells and lymphocytes.

While the canonical function of telomerase includes the inherent RNA component (TR/TERC) with the template region for telomere synthesis, during evolution novel properties of the TERT component have developed that are independent of the RNA and telomeres. These are usually called “non-canonical”. There have been various such non-canonical functions described (reviewed in [9,10,11] and many others) but their mechanisms of action are poorly understood. One of these non-telomeric functions consists in the ability of the TERT protein to shuttle upon oxidative stress from the nucleus to mitochondria where it can decrease ROS (reactive oxygen species) levels, cellular sensitivity to different drugs and apoptosis sensitivity [12,13]. In addition, there are various other processes where the TERT protein can be involved without its catalytic activity, for example in promoting tumourigenesis, epithelial-to mesenchymal transition (EMT) and DNA repair [14,15,16]. It can also interact with signalling pathways such as NF-KB, Myc and Wnt [17,18] as well as influence chromatin status [19].

### Subcellular Shuttling and Mitochondrial TERT

While for telomere maintenance TERT has to be localised in the nucleus, there are other possible subcellular localisations of the protein. Haendeler and colleagues demonstrated that for nuclear export, the TERT protein needs to be phosphorylated at tyrosine 707 by Src kinase in a Ran GTPase-dependent manner [20]. Increased oxidative stress seems be the most frequent trigger for nuclear export of TERT [12,13,21], but other stimuli such as rapamycin can have a similar effect [22]. Subcellular shuttling requires diverse signalling sequences, like the nuclear localisation signal (NLS), nuclear exclusion signal (NES) or the mitochondrial localisation signal (MLS) [21,23,24] The mitochondrial signalling/leader sequence of TERT is not present in organisms like plants and fungi (including yeast) but is exclusively present in higher eukaryotes such as mammals [21]. This is a good example for the acquisition of novel properties for telomerase/TERT during evolution.

Mitochondrial TERT localisation results in a decrease of cellular oxidative stress, reduced sensitivity to apoptosis and lower amounts of mitochondrial DNA damage in cancer cells or hTERT-overexpressing cells [12,13,25]. Surprisingly, in addition to decreased mitochondrial DNA damage, a decrease in nuclear DNA damage has been reported in cells with a mitochondrial TERT localisation [25]. In contrast, cells with a predominantly nuclear TERT localisation showed higher amounts of nuclear DNA damage upon increased stress. The underlying mechanism of this phenomenon is not well understood. It has been speculated that under conditions of oxidative stress, nuclear TERT could possibly interfere with DNA repair mechanisms [16,26,27]. Mitochondrial TERT is also able to upregulate antioxidant enzymes [10,28,29] thereby contributing to changes in cellular redox balance. Moreover, an improvement of cellular respiration by mitochondrial TERT localisation has been suggested as a possible mechanism of decreased ROS generation within mitochondria [13]. 

Another function of mitochondrial TERT protein is the ability to bind various mitochondrial DNA and RNA species [13,30]. By using certain mitochondrial t-RNAs as synthesis template, TERT acquires novel biochemical properties as a reverse transcriptase [30]. However, the biological consequences of this intriguing new property of telomerase within mitochondria remain elusive.

## 3. Telomerase and TERT in Brain

The first group to recognise and investigate the role of telomerase/TERT in neuronal cells was Mark Mattson’s. This group pioneered research on telomerase in brain using cultured pheochromocytoma cells and embryonic hippocampal neurons subjected to overexpression and knockdown of TERT. The resulting modifications to telomerase activity demonstrated the importance of telomerase against apoptosis and excitotoxicity [31,32]. At this time, around two decades ago, telomerase was known mainly for its canonical, telomere-maintaining function. Consequently, these early studies did not discriminate between canonical and non-canonical functions of telomerase/TERT. Nevertheless, they already described a protective function of telomerase/TERT both during brain development [33] and also for neurodegeneration by treating neuronal cultures with amyloid-β (Aβ) [34]. The group also demonstrated the decline of telomerase activity in mouse brain after embryonic day 13 and its disappearance by postnatal day 10 [35]. Conversely, *mTert (mouse Tert)* expression was maintained into adulthood, suggesting non-telomeric functions of the protein. Telomerase activity in the brain is mainly associated with neural stem cells in certain areas such as the subventricular zone, the hippocampal dentate gyrus, and a few other regions [36,37]. However, while most neurons in the brain have no telomerase activity [35,38], there are exceptions such as Purkinje neurons which were shown to have telomerase activity and TERT protein in the nucleus as well as the cytoplasm [39,40]. In general, there has not been a systematic analysis of possible differences in telomerase biology and localisation of the TERT protein in different types of neurons or in different brain regions.

The mechanism of downregulation of telomerase activity in the brain seems to be different from other somatic tissues. While in human somatic tissues the amount of TERT protein is an important regulator and limiting factor for telomerase activity (TA), the RNA subunit TERC in these tissues is constitutively expressed. In contrast, in human brain, the hTERC component is downregulated at very early stages of development (after postconception week 10) [38] and thus is most likely the factor responsible for the absence of telomerase activity at later stages of development and in adult brain tissue. However, TERT persists in neurons and the brain [40,41,42]. Timing of downregulation of telomerase activity seems to be different in mouse brain where telomerase activity is active until early postnatal stages [35]. Additionally, *mTert* expression in mouse brain seems to be downregulated at higher ages [22] while no systematic study has yet been performed on a possible decline in human brain tissue. These differences between mouse and human brain are not surprising, considering the striking differences in telomere and telomerase biology between these two species while other mammals might have different specificities [43]. 

Intriguingly, Esther Priel’s group has demonstrated the existence of an alternative mTERC component (alTERC) lacking 18 nucleotides in the conserved CR4 region in mouse brain, spleen and motor neuron cells which also protects against oxidative stress [44]. However, it is not known yet whether this type of RNA also exists in human brain. Interestingly, the authors demonstrated that the alternative TERC was able to bind both mouse and human TERT protein and to form enzymatically active telomerase in vitro [44].

Several groups have shown that adult mammalian hippocampal neurons maintain TERT protein without telomerase activity [41,42]. This also fits well with its non-nuclear localisation in these neurons [41,42]. However, this could be different in other neuron types since telomerase activity was described in mouse Purkinje neurons but not in the granular and molecular layer of the cerebellum [40]. The authors found TERT localisation in the nucleus, cytoplasm and mitochondria, which could be specific to Purkinje neurons and possibly also to mice. External stress such as γ-irradiation in vivo and high glutamate concentration in situ on cerebellar slices increased TERT protein levels in the nucleus and mitochondria, respectively [40]. Furthermore, it has been shown that human astrocytes are not positive for TERT protein [41] but are able, at least in rodents, to activate *Tert* expression transiently after brain injury [45,46]. In contrast, activated human microglia cells are positive for TERT protein even in brains from old subjects [41]. However, in culture, rodent-derived microglia and astrocytes independently of possible low levels of telomerase activity shorten their telomeres and enter senescence [47].

The subcellular localisation of TERT protein outside the nucleus in hippocampal neurons [41,42] differs from most mitotic cell types where it is found predominantly in the nucleus performing its canonical function on telomeres. Neurons as postmitotic cells do not divide and thus do not shorten their telomeres but may still accumulate telomeric DNA damage, which can result in a senescent phenotype [48,49]. Other studies also emphasised the importance of the senescence-associated secretory phenotype (SASP) in non-neuronal cells for brain ageing, cognitive decline and neurodegenerative diseases [50,51]. Senescence can be caused by telomere dysfunction due to the absence or age- and disease-related decrease of telomerase activity.

Interestingly, Iannilli and co-authors discovered that cytoplasmic TERT protein in mature mouse hippocampal neurons is able to form complexes with RNA particles and binds the RNA of the cyclin-dependent kinase inhibitor p15Ink4b in the cytoplasm [42]. The authors demonstrated that upon stress the complex is resolved, and the different components are free to perform different functions. They also suggested that at this stage TERT protein might be able to enter mitochondria. Thus, it is not clear where exactly TERT resides in unstressed neurons—whether in the cytoplasm or whether a certain fraction is always localised within mitochondria.

Due to the absence of good TERT antibodies for mouse TERT [52] which would also work specifically in brain tissue of this species, it is rather difficult to analyse the subcellular TERT protein localisation in rodent brain tissue directly and in detail.

## 4. TERT and Neurodegenerative Diseases

Many neurodegenerative diseases such as AD and PD can be either caused genetically by mutations or occur spontaneously as in the majority of cases. There are many facets to brain ageing and the underlying causes of age-related neurodegenerative diseases are not well understood yet and thus no cures are available. However, among the most likely candidates contributing to those diseases are oxidative stress, mitochondrial dysfunction as well as the accumulation of toxic proteins such as amyloid-β, pathological tau and α-synuclein [53]. With increasing evidence of a potential role of telomerase and the TERT protein in the brain, various groups started to analyse different models in order to evaluate a possible beneficial role of telomerase and TERT.

The TERT protein has been demonstrated to provide a protective function for neurons against various stimuli such as oxidative stress, high glutamate and neurodegenerative agents such as amyloid-β and pathological tau [40,41,54]. Neurons lacking TERT protein due to a genetic knock-out develop higher amounts of oxidative stress after being transduced with pathological tau [41].

Since Alzheimer’s disease occurs predominantly at higher age, one would expect that neuronal TERT protein levels might decrease during disease progression. However, a study from the Saretzki group on hippocampal tissue from AD brains and age-matched controls did not find a decrease of TERT protein during the development of AD at different Braak stages [41]. Instead, the study found a higher amount of TERT protein in mitochondria of hippocampal CA1 neurons at the highest Braak stage compared to healthy aged-matched controls. However, it is not understood whether mitochondrial TERT localisation is a consequence of higher oxidative stress which is known to be associated with AD [53] or whether mitochondrial TERT really protects those neurons from stress.

Intriguingly, the study also revealed that pathological tau protein in the form of neurofibrillary threads and tangles in hippocampal neurons at higher Braak stages seems to be mutually exclusive with TERT protein [41]. However, it is not clear whether pathological tau in AD brains displaces TERT protein during disease progression or whether neurons with high amounts of TERT protein are protected from disease-associated tau. In order to address this question more mechanistically, the study used isolated and cultured primary embryonic mouse neurons at a stage when they were not positive for telomerase activity anymore and transduced them lentivirally with mutated tau (p301L) [41]. Employing neurons from wild-type and *Tert* knockout (KO) mice [55], the authors demonstrated clear differences in oxidative stress (higher levels of reactive oxygen species ROS and lipid peroxides) as a result of the p301L transduction [41]. These results suggest that TERT protein might indeed protect neurons from the damaging effects of pathological tau.

Regarding a role for senescence in non-neuronal cells in AD, Bussian and co-authors showed that selectively removing senescent astrocytes and microglia cells with senolytics improved AD-related symptoms in a genetic mouse model of AD expressing the p19S tau mutation [51]. Although telomerase was not analysed specifically in this study, the results confirm that many different cell types in the brain participate in phenotypes related to neurodegeneration.

A potential role of TERT in PD will be discussed in the next section in conjunction with the use of telomerase activators.

## 5. Increasing Telomerase/TERT Levels in Ageing Brain and as Therapy for Neurodegeneration

The establishment of a protective effect of telomerase in brain and neurons has prompted several groups to employ experimental interventions in order to increase TERT levels. This could benefit brain function like cognition during ageing as well as pose a therapeutic option for treating neurodegenerative diseases.

### 5.1. TERT-Overexpression

Wittemore and co-authors [56] increased telomerase in late generation *Terc* KO mice with short telomeres using adeno-associated viruses [57]. This treatment was able to ameliorate some age-related memory deficits in the mouse model which, in the author’s opinion, resembles neurodegenerative phenotypes of diseases like Alzheimer’s disease. The result suggests that short telomeres can contribute to brain ageing and neurodegenerative symptoms and that increasing the telomerase/TERT levels in mouse brain had a significant effect on the decrease of cognitive impairment which is an important hallmark of neurodegenerative diseases. The authors found more neurogenesis but less inflammation and DNA damage in the brains with increased telomerase without specifying the different cell types. Thus, possibly together with stabilising telomeres, telomerase might also reduce the amount of senescence, including a decreased SASP, as the importance of those parameters has been described previously for different brain cell types including Purkinje neurons [48,49,50,51].

### 5.2. The Use of Telomerase Activators to Boost TERT Levels in Brain and in Models of Neurodegeneration

In addition to genetic interventions as described above, both, natural and synthetic telomerase activators have been employed in order to boost TERT levels in the brain. However, the exact mechanism by which these activators promote telomerase activity and TERT levels is not well understood. They could act via an increase of *TERT* transcription by inducing transcription factors and signalling pathways. For example, for TA-65, a plant-derived cycloastragenol, an induction of kinases such as ERK has been suggested [58]. With *TERT* expression being an important regulator of telomerase activity, the latter can be activated in cell and tissue types that are proficient for TA. Different cellular models have been used to confirm increased telomerase activity and *TERT* expression after treatment with different telomerase activators: late-generation MEFs (mouse embryonic fibroblasts) from TERC heterozygous mice, HEK cells, cultured primary cortical and hippocampal neurons or bone marrow cells [58,59,60]. In addition, measurement of telomere elongation, in particular that of the shortest telomeres, suggests the action of telomerase activators on canonical telomerase activity [61,62]. In contrast, in tissues without or with rather negligible telomerase activity, the expression of *TERT* seems to be a valid method of analysing telomerase-related effects of the activators.

#### 5.2.1. Synthetic Aryl Compounds as Telomerase Activators

Esther Priel’s group was the first to use a synthetic telomerase activator (aryl compound, AGS-499) directly on a brain related disease: Amyotrophic Lateral Sclerosis (ALS). They demonstrated an improvement of disease scores in a mouse model of ALS using subcutaneous injection of the compound and suggested a better survival of affected motor neurons as underlying mechanism [63]. A beneficial role of telomerase in ALS was also confirmed by a study which demonstrated that telomerase knock-out resulting in progressive telomere shortening, accelerated the ALS phenotype in a transgenic SOD1 (the gene mutated in ALS) mouse model [64]. In addition, Eitan et al. also showed a protective effect in brains of AGS treated mice against NMDA-induced cytotoxicity [63]. The authors confirmed the specificity of the AGS compound by demonstrating a lack of a protective effect on viability and DNA damage in telomerase-inhibited cells after application of oxidative stress.

In a recent study, the same group used cultured primary hippocampal mouse cells consisting of both neurons and astrocytes and exposed them to amyloid-β in order to create a cellular model for AD [54]. Treatment with Aβ induced neurotoxicity and compromised gene expression of factors involved in neuronal survival and plasticity. In contrast, the increase of TERT levels with the synthetic telomerase activator showed protection from the toxic effects of Aβ and the resulting neuronal damage [54]. The authors identified the involvement of the Wnt/beta-catenin pathway and showed increased expression of neurotrophic factors such as BDNF and NGF as well as neuronal plasticity genes to be the underlying mechanisms of protection by TERT [54].

#### 5.2.2. Beneficial Health Effects of Cycloastragenol-Based Telomerase Activators

There are currently two more telomerase activators under investigation: TA-65 and GRN510 from TA Science Inc. (USA). TA-65 is a highly purified plant extract from the Mongolian milkvetch (*Astragalus membranaceus*) which has been demonstrated previously to extend the shortest telomeres in blood lymphocytes from mice and humans when used orally as part of the diet [58,61,62]. Maria Blasco’s group administered TA-65 to adult and aged female mice for 3 months and found an elongation of the shortest telomeres in peripheral blood monocytes (PBMCs) as well as an improvement of various health parameters such as glucose tolerance and skin properties during ageing without an increase in cancer incidence, although lifespan was unchanged [58]. These results are similar to another study of the same group which used adeno-associated viruses (AAV) to increase telomerase in one- and two-year-old mice [57]. This intervention also improved health parameters such as insulin sensitivity, neuromuscular coordination and cognition as well as other biomarkers of ageing. Again, no increased cancer incidence was detected, but this time, the intervention also increased median lifespan [57]. A possible reason for the differences in lifespan could be a more systemic increase of *Tert* expression in all analysed tissues (including brain) [57] compared to the 3 months treatment with TA-65 where only liver showed a significant increase of *Tert* expression [58]. Importantly, the authors of the TA-65 study also demonstrated an increase in telomere length and decrease of DNA damage in mouse embryonic fibroblasts (MEFs) from heterozygous TERC KO mice while no such effect was found in MEFs from late generation homozygous TERC KO mice [58]. Obviously, the effect of the telomerase activator on telomeres was predominantly dependent on the increase of enzymatic telomerase activity. However, regarding DNA damage the study only analysed general damage. It would be interesting to determine how much of this damage was related to and localised at telomeres. Additionally, in the light of differential sensitivity of various brain cell types, it would be interesting to discriminate between the DNA damage in neurons and non-neuronal cells.

Clinical studies using TA-65 demonstrated an amelioration of symptoms of age-related diseases. For example, it was able to ease pain in the eyes and improve vision in human patients with macular degeneration [65]. Others employed TA-65 in patients with metabolic syndrome and found improved cardiovascular parameters such as cholesterol (HDL) and substantially decreased inflammation in the blood [66]. Unfortunately, these clinical studies did not measure telomere length in their patients. Consequently, it is difficult to judge whether it were mainly telomere-related effects which played a role in the improvements or whether non-telomeric functions of TERT also contributed. In general, the activator was previously used primarily with the intention of enhancing telomerase activity for the extension of critically short telomeres and thereby acting as an anti-ageing intervention.

The synthetic analogue of the plant cycloastragenol, GRN510, was used in a model of pulmonary fibrosis where it activated telomerase activity in haematopoietic progenitor cells ex vivo as well as in bone marrow and lung epithelial cells in vivo [60]. However, brain tissue was not analysed in this study.

A recent study by Wan and colleagues applied both activators, TA-65 and GRN510, in a pilot study on two-year-old female mice for 3 months which resulted in a significant increase of *mTert* expression in brain tissue [67]. Analysing motor coordination as a read-out for age-related brain function, the study found a significant improvement of strength and coordination in a static rod test where both treatments increased the time on the rod similarly to that of adult (10 months old) mice while only that for GRN510 was statistically significant [67]. These results are similar to those described by Eyolfson and co-authors who treated rats for one month with TA-65 before inflicting a brain injury. The authors found an increase in *Tert* expression as well as an improvement in motor coordination in the treated rats compared to controls [68].

#### 5.2.3. Effects of Telomerase Activators on a Mouse Model of Parkinson’s Disease (PD)

Until recently, not much was known about a possible role of TERT or telomerase in PD. Vera and co-workers used human iPS (induced pluripotency stem) cells to address the role of telomeres in an *in vitro* disease model. Inhibiting telomerase activity in the iPS cells generated midbrain (dopaminergic) neurons with shorter telomeres during differentiation which displayed age-associated and disease-related phenotypes [69].

A recently published study from the Saretzki group used TA-65 and GRN510 in a transgenic mouse model of Parkinson’s disease (PD). This model was generated by Elizier Masliah and overexpresses human wild-type α-synuclein under a PDGF promoter [70]. The mice accumulate α-synuclein in the hippocampus, neocortex and olfactory bulb at an age of around one year and have decreased levels of dopamine and tyrosine hydroxylase in the substantia nigra [71]. In addition to PD-like pathology, the mice show behavioural deficits which resemble motor symptoms of PD [70,71]. The fact that α-synuclein accumulation takes around one year to peak in the transgenic mouse model strongly suggests an age-dependency of disease progression similar to that of many neurodegenerative diseases.

Both activators significantly increased *mTert* gene expression in both sexes after 14 months of treatment starting at 4 months. Additionally, *in vitro* experiments on cultured primary embryonic mouse neurons at a time point where these neurons were no longer positive for telomerase activity [41] confirmed specifically that neurons were able to upregulate *mTert* expression after activator treatment [67].

Since impaired motor coordination is an important symptom for PD, behavioural tests for motor activity such as the rotarod test, gait test and walking speed were employed to characterise treatment effects [67]. Interestingly, results from the rotarod test showed some striking sex specificities: in females only TA-65 improved motor parameters, in males a significant improvement was achieved exclusively with GRN510. Others have described similar effects with TA-65 previously and speculated about the involvement of sex hormones such as oestrogen [69] which is involved in *TERT* transcriptional regulation. However, in the Wan et al. study, other tests measuring walking speed for bradykinesia and gait identified no such sex-differences. Gait analysis measures stride length and width as well as variations therein and is similar to clinical tests used on PD patients [72]. Both activators greatly improved the walking speed as well as the length and width of strides, with a reduction in stride variation [67].

In order to identify possible mechanisms for TERT’s protective effects, the authors measured forward and reverse electron flow in brain mitochondria but found an improvement only for TA-65 but not for GRN510. Possibly the more heterogenous nature of the natural plant extract, which is 95% pure but also contains around 5% other compounds [58,61], could explain this difference.

**Figure 1:**
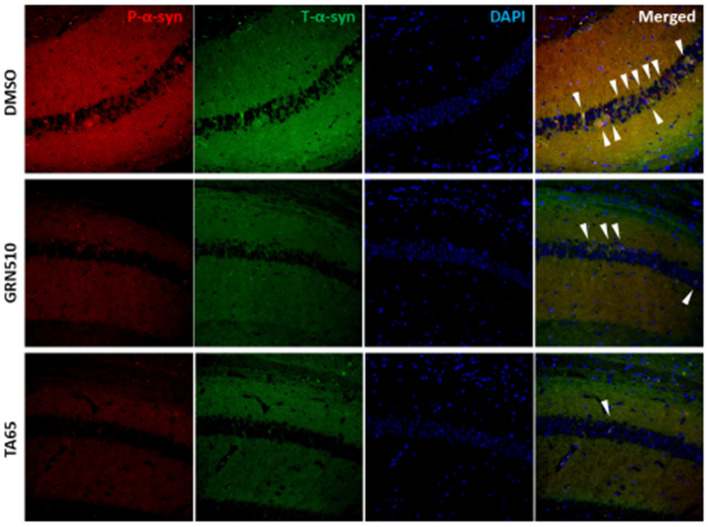
Phosphorylated (red signal) and total (green signal) α-synuclein in the CA1 region of the hippocampus of DMSO-treated (control), GRN510 and TA-65 treated mice. White arrowheads show merged signals of phosphorylated and total α-synuclein. Blue signals are DAPI for nuclear staining. For details and quantification, see [67].

In addition to behavioural tests, the study of Wan and co-authors analysed the levels of various forms of α-synuclein in brains from the PD mouse model. Intriguingly, they found a striking decrease of total, phosphorylated (S129) as well as aggregated α-synuclein in the three analysed brain regions: hippocampal regions CA1 and CA3 as well as neocortex. Both activators decreased total α-synuclein levels, while only TA-65 decreased phosphorylated α-synuclein levels in all three regions significantly [67]. Figure 1 shows representative images of total and phosphorylated α-synuclein in the hippocampal CA1 region for all 3 treatment groups.

**Figure 2:**
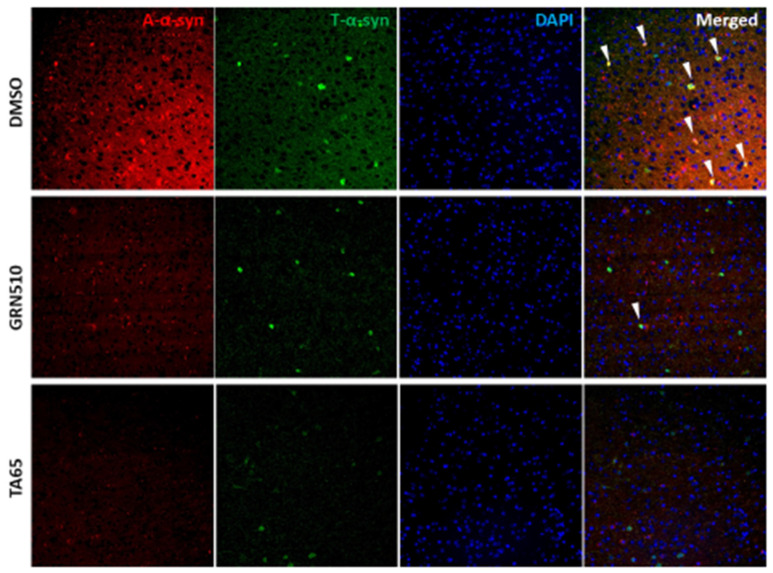
Aggregated (red signal) and total (green signal) human α-synuclein in the neocortex of DMSO-treated (control), GRN510 and TA-65 treated mice. The white arrowheads show a yellow merged signal of neurons with aggregated on top of total α-synuclein. Blue DAPI staining of nuclei. For details and quantification, see [67].

Importantly, aggregated α-synuclein was decreased significantly by both activators in all three brain regions [67]. Figure 2 shows examples for aggregated and total α-synuclein in the neocortex. As expected for postmitotic neurons, the study did not detect any changes in telomere length in neurons from the hippocampal region CA1. In contrast, Wittemore and colleagues measured telomere length only in blood cells where they detected an increase after enhancing telomerase but found a decrease of DNA damage in various brain regions due to increased telomerase using adenoviral transduction of TERT [56]. However, they did not analyse whether DNA damage occurred evenly in the genome or whether telomeres were targeted predominantly. Likewise, they did not discriminate between brain cell types for DNA damage; thus, it is not clear how much neurons were involved in the improvement or whether it was primarily due to non-neuronal cell types.

## 6. TERT, mTOR and Autophagy in Brain

mTOR is a conserved serine/threonine kinase consisting of two protein complexes: mTORC1 and mTORC2. The mTOR pathway regulates cell growth and metabolism in response to nutrient and energy supply. mTOR is an important regulator of autophagy. Both, mTOR and autophagy are critically involved in brain physiology and are increasingly recognised as important players in neurodegeneration [73,74,75]. Autophagy is prominently involved in the degradation of toxic brain proteins such as misfolded and aggregated α-synuclein in PD [75]. Another mechanism of protein quality control is proteasomal degradation of monomeric and oligomeric toxic proteins in the brain [76].

Two papers recently provided evidence for an involvement of telomerase in promoting these two protein degradation mechanisms in cellular models [77,78]. Im and co-authors demonstrated that overexpression of *hTERT* promoted assembly of proteasomal subunits and functional improvement of proteasomal degradation in a cancer cell line [77]. Ali et al. described a decrease of mTOR activity through mTORC1 due to overexpression of *hTERT* in human cancer cells which resulted in an increase in autophagy [78]. In contrast, cells lacking *TERT* were unable to execute autophagic flux by autophagosomes.

There have been previous hints of a possible functional as well as physical interaction of TERT with mTOR. Interestingly, these two molecules seem to form a physical complex together with other molecules both in immune cells and cancer cells [79,80].

The first *in vivo* brain model relating to TERT and mTOR was described by Miwa and co-authors in connection with the effects of dietary restriction and rapamycin treatment, both involving decreases in the mTOR pathway resulting in increased autophagy. The authors demonstrated that TERT interacts functionally with the mTOR pathway. Only brain tissue from wild-type mice showed a decrease in ROS levels after rapamycin treatment (which decreases mTORC1) while in *Tert* KO mice this effect was abolished [22]. In an *in vitro* cellular model rapamycin induced a subtle exclusion of a small fraction of TERT protein from the nucleus which correlated to a decrease in ROS levels [22]. In contrast, blocking nuclear TERT exclusion with a src inhibitor or using *TERT*-knock-out cells abolished the effect of rapamycin in decreasing ROS. Importantly, the study demonstrated the dependence of ROS reduction by rapamycin specifically in the brain but not in liver of wild-type mice while the effect was abolished in *Tert* knockout mice [22].

Wan et al. explored in their study whether autophagy was involved in the effect of telomerase activators on the decrease of α-synuclein in the PD mouse model [67]. The authors analysed the two autophagy parameters p62 (an adaptor protein which decreases when autophagy is induced) and LC3B (a membrane-bound molecule of the autophagosome which decreases when the turnover of these organelles is increased). Both p62 and LC3B decreased significantly in the hippocampal CA1 due to telomerase activator treatment [67]. These results suggest that increased TERT levels due to a treatment with telomerase activators might be able to increase protein quality control by autophagy to degrade different α-synuclein forms in the brain, although the authors did not analyse specifically whether the mTOR pathway or increased proteasomal degradation were involved as well. However, Crews et al. demonstrated previously in the same mouse model of PD that mTOR and LC3B were increased compared to wild-type mice and importantly, both markers co-localised with α-synuclein [81]. The authors treated the transgenic mice with rapamycin which decreases mTOR activity and found an increase in autophagy and decrease in aggregated α-synuclein. This result by Crew et al. emphasises the capability of autophagy to degrade aggregated α-synuclein without studying telomerase/TERT as a possible factor.

## 7. Conclusions

There is increasing interest in the role of telomerase for brain function, such as cognitive ability, ageing, brain injury as well as neurodegenerative diseases. Various studies have demonstrated a protective function of telomerase/TERT protein in neurons against toxic neurodegenerative proteins including amyloid-β, pathological tau and α-synuclein [41,54,67]. However, the mechanisms for that protection are not entirely clear. Some studies have identified mitochondrial TERT localisation as well as autophagy and the mTOR pathway as possible mechanisms through which TERT might exert its protective function in brain and neurons [22,41,67] while other groups have found specific growth factors and genes involved in brain plasticity to be stimulated by increased TERT levels [54]. The results in a preclinical mouse model of PD are encouraging and suggest that the use of the nutraceutical TA-65 might alleviate PD-related symptoms. This could result in the development of novel therapeutic strategies for the treatment of PD and other neurodegenerative diseases. Studies from various groups using either telomerase activators or other experimental approaches such as adenoviral overexpression of *TERT* for the increase of TERT levels in brain support this strategy [54,56,57].

Thus, clinical trials with early stages of PD patients or other neurodegenerative diseases such as AD are required to confirm the beneficial effects of increasing telomerase/TERT levels in brains of patients suffering from neurodegenerative diseases. Moreover, due to the already known anti-ageing effects of TA-65 on other organs and cell types (for example blood lymphocytes), such a treatment could also improve different age-related and telomere-dependent health parameters. In addition, preventing senescence and SASP-induced inflammation in various types of brain cells such as neurons, astrocytes and microglia [48,49,50,51,82] could be another important mechanism to protect the brain from age-related decline of cognition as well as to ameliorate the development or progression of neurodegenerative diseases. As those diseases pose an increasing health problem and socio-economic burden for societies all over the world, there is increasing demand for novel therapeutic interventions. Boosting telomerase/TERT level in brain could empower novel treatment strategies for these diseases in the future.

**Authors’ contribution:** G.S. conceptualised and wrote the review; T.W. composed the images, reviewed and approved the manuscript. All authors have read and agreed to the published version of the manuscript.

## Data Availability

Not applicable.
